# The Effects of Synthetic Oligopeptide Derived from Enamel Matrix Derivative on Cell Proliferation and Osteoblastic Differentiation of Human Mesenchymal Stem Cells

**DOI:** 10.3390/ijms150814026

**Published:** 2014-08-13

**Authors:** Nobuhito Katayama, Hirohito Kato, Yoichiro Taguchi, Akio Tanaka, Makoto Umeda

**Affiliations:** 1 Department of Periodontology, Osaka Dental University, Osaka 573-1121, Japan; E-Mails: taguchi@cc.osaka-dent.ac.jp (Y.T.); umeda-m@cc.osaka-dent.ac.jp (M.U.); 2 Department of Oral Pathology, Osaka Dental University, Osaka 573-1121, Japan; E-Mails: kato-h@cc.osaka-dent.ac.jp (H.K.); tanaka@cc.osaka-dent.ac.jp (A.T.)

**Keywords:** human mesenchymal stem cells, oligopeptides, cell differentiation, enamel matrix derivative

## Abstract

Enamel matrix derivative (EMD) is widely used in periodontal tissue regeneration therapy. However, because the bioactivity of EMD varies from batch to batch, and the use of a synthetic peptide could avoid use from an animal source, a completely synthetic peptide (SP) containing the active component of EMD would be useful. In this study an oligopeptide synthesized derived from EMD was evaluated for whether it contributes to periodontal tissue regeneration. We investigated the effects of the SP on cell proliferation and osteoblast differentiation of human mesenchymal stem cells (MSCs), which are involved in tissue regeneration. MSCs were treated with SP (0 to 1000 ng/mL), to determine the optimal concentration. We examined the effects of SP on cell proliferation and osteoblastic differentiation indicators such as alkaline phosphatase activity, the production of procollagen type 1 *C*-peptide and osteocalcin, and on mineralization. Additionally, we investigated the role of extracellular signal-related kinases (ERK) in cell proliferation and osteoblastic differentiation induced by SP. Our results suggest that SP promotes these processes in human MSCs, and that ERK inhibitors suppress these effects. In conclusion, SP promotes cell proliferation and osteoblastic differentiation of human MSCs, probably through the ERK pathway.

## 1. Introduction

Periodontal disease is a chronic inflammation that destroys the periodontal tissue [1]. Various therapies for regenerating periodontal tissue destroyed by periodontal disease have been tested. Some materials that have been used in periodontal tissue regeneration therapy include bone morphogenetic protein-2 (rhBMP-2) [2], platelet-derived growth factor (PDGF) [3], and enamel matrix derivative (EMD) [4]. EMD is composed of amelogenin, ameloblastin and tuftelin, and is produced by extraction from the tooth buds of juvenile swine [5]. EMD is widely used in periodontal tissue regeneration therapy, because it induces the regeneration of periodontal tissue lost in periodontal disease [6,7]. EMD promotes cell proliferation, osteoblastic differentiation and mineralization in mesenchymal stem cells (MSCs), periodontal ligament (PDL) cells, osteoblasts and cementoblasts, all of which are cells involved in periodontal tissue regeneration [8,9]. Although EMD clinically promotes periodontal tissue regeneration, this substance is derived from animal tissue and therefore contains unknown pathogens and a mixture of heterogeneous proteins [10]. Additionally, the activity of EMD can differ depending on lot number. Therefore, to best use the clinical data obtained regarding the regeneration effects and postoperative stability of EMD, a completely synthetic peptide containing the active component of EMD is required.

In our previous study, we found that EMD administered underneath the skin on the backs of rats induced cartilage-like tissue formation and eosinophilic round bodies (ERBs) [11]. Analysis of the active sites for ERB binding in EMD-associated proteins was conducted using matrix-assisted laser desorption ionization time-of-flight mass spectrometry. This database analysis identified a seven amino acid sequence (WYQNMIR) in amelogenin that corresponds to a portion of the amelogenin exon 5 [11]. Based on this sequence, a synthetic oligopeptide (SP) was constructed.

EMD has been reported to be antigenic and to induce the production of anti-EMD antibodies in the host [12]. Shinnick *et al.* and Lerner have suggested that only peptides of greater than ~10 residues (or over 5 kDa) can function as antigens [13,14]. Thus, because SP contains only seven amino acids and its molecular mass is 1118 kDa, it is less likely to elicit an immunological response, compared with EMD.

We found that SP induced bone formation underneath the skin of rats [15], and that the regeneration of alveolar bone tissue was stimulated in artificial periodontal defects similarly to EMD [16]. Moreover, we have reported that SP promotes cell proliferation of human PDL fibroblasts [17] and rat bone marrow stromal cells [18]. SP also induced osteoblastic differentiation and hard tissue formation of PDL fibroblasts [19] and PDL stem cells [20]. These findings may contribute to the formation of new hard tissue and connective tissue, which is essential for periodontal tissue regeneration.

MSCs are highly proliferative and have a high potential to undergo osteoblastic differentiation, and thus contribute to the regeneration of various tissues [21]. MSCs also contribute to periodontal tissue regeneration to induce the formation of alveolar bone and PDL tissue. Previous studies have reported that transplantation of MSCs contributes to periodontal tissue regeneration [22,23,24]. However, no studies have demonstrated the effects of SP on human MSCs. Therefore, this area of investigation is meaningful.

Mitogen-activated protein kinases (MAPKs) are important regulators of cell proliferation and osteoblastic differentiation [25]. The MAPK family comprises extracellular signal-related kinases (ERK) 1/2, p38 MAPKs (p38), and Jun amino-terminal kinases (JNK) [26]. Among the three different MAPK signaling molecules, ERK 1/2 regulates the cell proliferation and osteoblast differentiation induced by EMD and amelogenin peptides [27,28]. However, it is not clear whether SP induces the effects of this signaling pathway on cell proliferation and osteoblastic differentiation.

The aim of this study was to investigate the effect of SP on cell proliferation and osteoblastic differentiation of human MSCs. Additionally, we examined ERK 1/2 signaling with regard to the interaction between SP and human MSCs using an ERK 1/2 inhibitor.

## 2. Results and Discussion

### 2.1. Results

#### 2.1.1. Cell Proliferation

**Figure 1:**
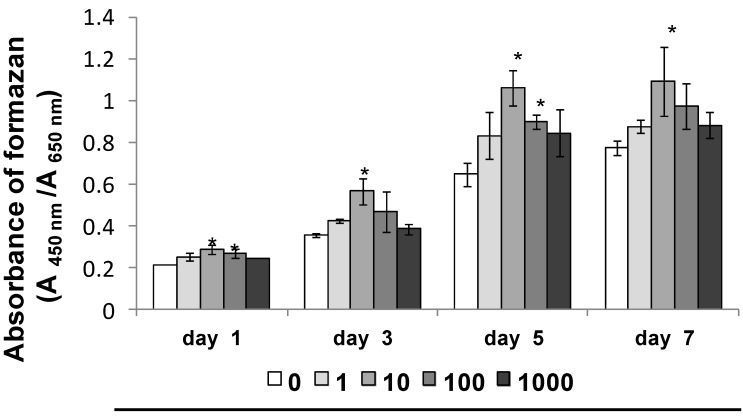
Effect of synthetic peptide (SP) on cell proliferation. mesenchymal stem cells (MSCs) were seeded in 96-well plates at 2 × 10^3^ cells/well in normal culture medium. After a 24-h culture for cell adherence, the medium was replaced with normal culture medium containing SP (0, 1, 10, 100 or 1000 ng/mL) and MSCs were incubated for 1, 3, 5 and 7 days. Cell proliferation was measured on days 1, 3, 5 and 7. * *p* < 0.05 *vs.* SP (0 ng/mL), at days 1, 3, 5 and 7.

SP significantly promoted cell proliferation of MSCs cultured for 1, 3, 5 and 7 days in normal culture medium (Figure 1, days 1, 3, 5 and 7 * *p* < 0.05). Additionally, SP promoted the highest cell proliferation at a concentration of 10 ng/mL.

#### 2.1.2. Alkaline Phosphatase (ALP) Staining and Measurement of ALP Activity

**Figure 2:**
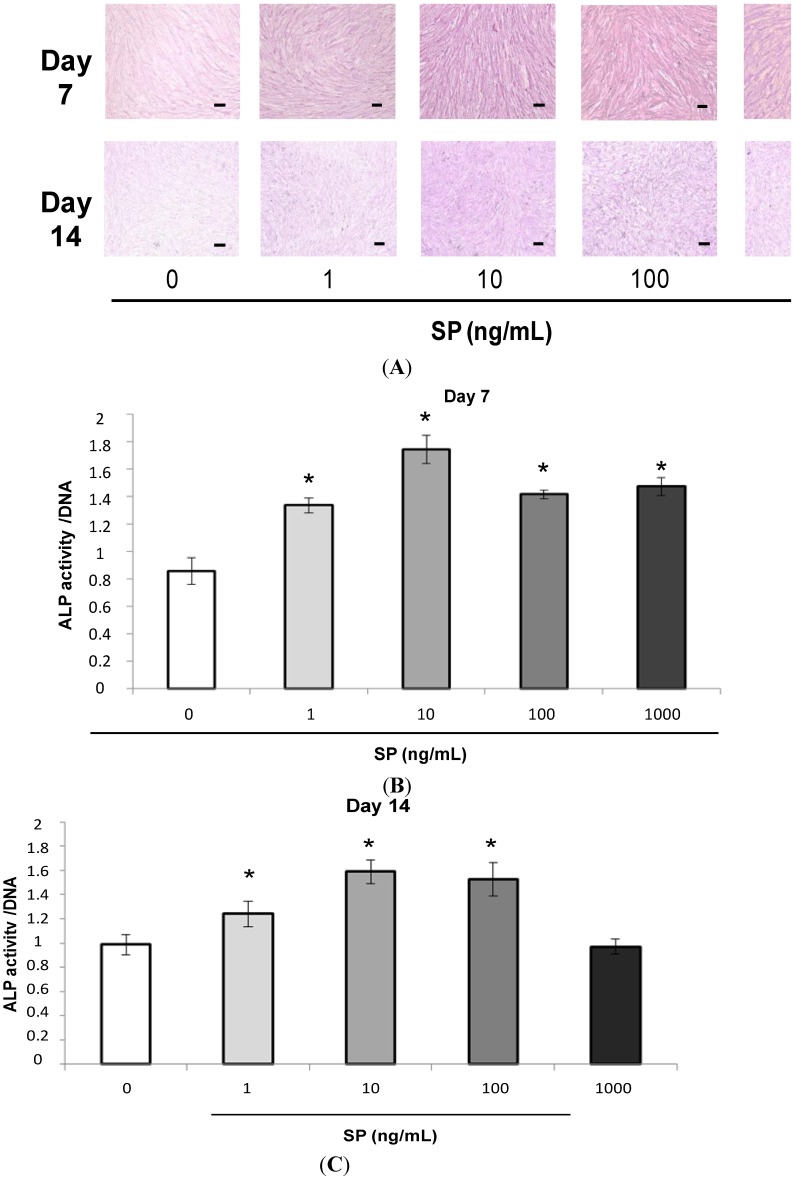
Effect of SP on alkaline phosphatase (ALP) staining and ALP activity. (**A**) Confluent MSCs were stained using an ALP staining kit after 7 or 14 days of cultivation in osteogenic medium containing SP (0, 1, 10, 100 or 1000 ng/mL). Scale bar = 100 μm; (**B**,**C**) ALP activity was measured at 7 or 14 days. To normalize ALP activity, the amount of ALP was normalized to the amount of DNA; (**B**) day 7, * *p* < 0.05 *vs.* SP (0 ng/mL); and (**C**) day 14, * *p* < 0.05 *vs.* SP (0 ng/mL).

The intensity of ALP staining was stronger in cultures containing SP (Figure 2A, days 7 and 14). SP also significantly promoted ALP activity in osteogenic medium at 7 and 14 days (Figure 2B day 7, and Figure 2C day 14, * *p* < 0.05). Additionally, SP promoted both the highest intensity of ALP staining and the highest ALP activity at a concentration of 10 ng/mL.

#### 2.1.3. Procollagen Type 1 *C*-Peptide (PIP) and Osteocalcin (OCN) Production

SP significantly promoted Procollagen Type 1 *C*-Peptide (PIP) and Osteocalcin (OCN) production in osteogenic medium on days 7 and 14 of culture (Figure 3A day 7, and Figure 3B day 14, * *p* < 0.05). Additionally, SP promoted the highest production of both PIP and OCN at a concentration of 10 ng/mL.

**Figure 3:**
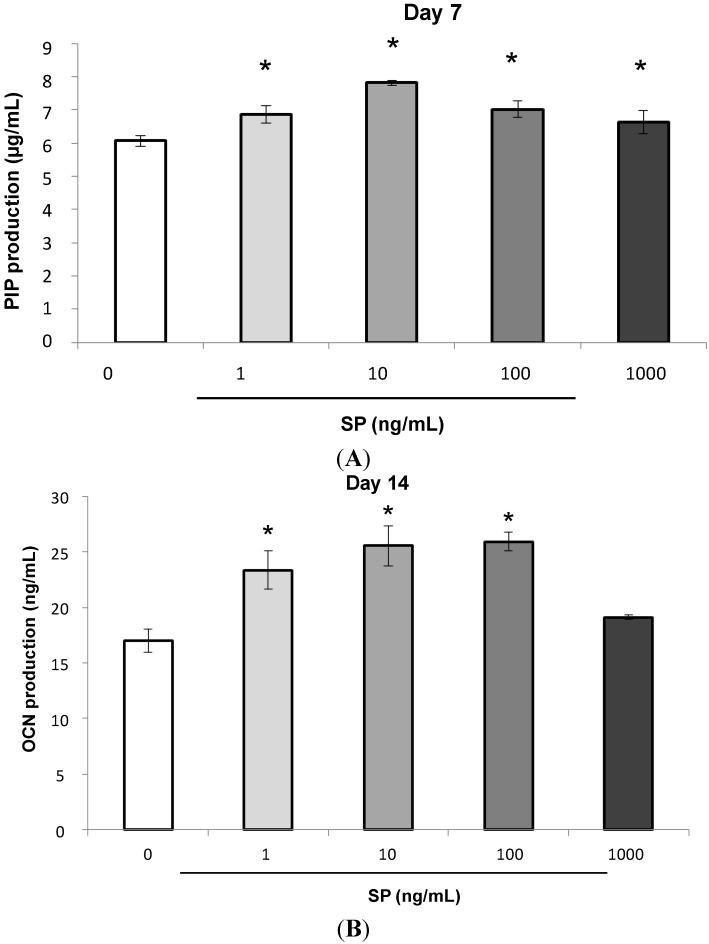
Effect of SP on the production of Procollagen Type 1 *C*-Peptide (PIP) and Osteocalcin (OCN). After the MSCs reached confluence, the culture medium was replaced with osteogenic medium containing SP (0, 1, 10, 100 or 1000 ng/mL) and the cells were cultured for 7 or 14 days. (**A**) day 7, * *p* < 0.05, *vs.* SP (0 ng/mL); and (**B**) day 14, * *p* < 0.05, *vs.* SP (0 ng/mL).

#### 2.1.4. Mineralization Assay (Alizarin Red Staining and Extracellular Calcium Deposition)

**Figure 4:**
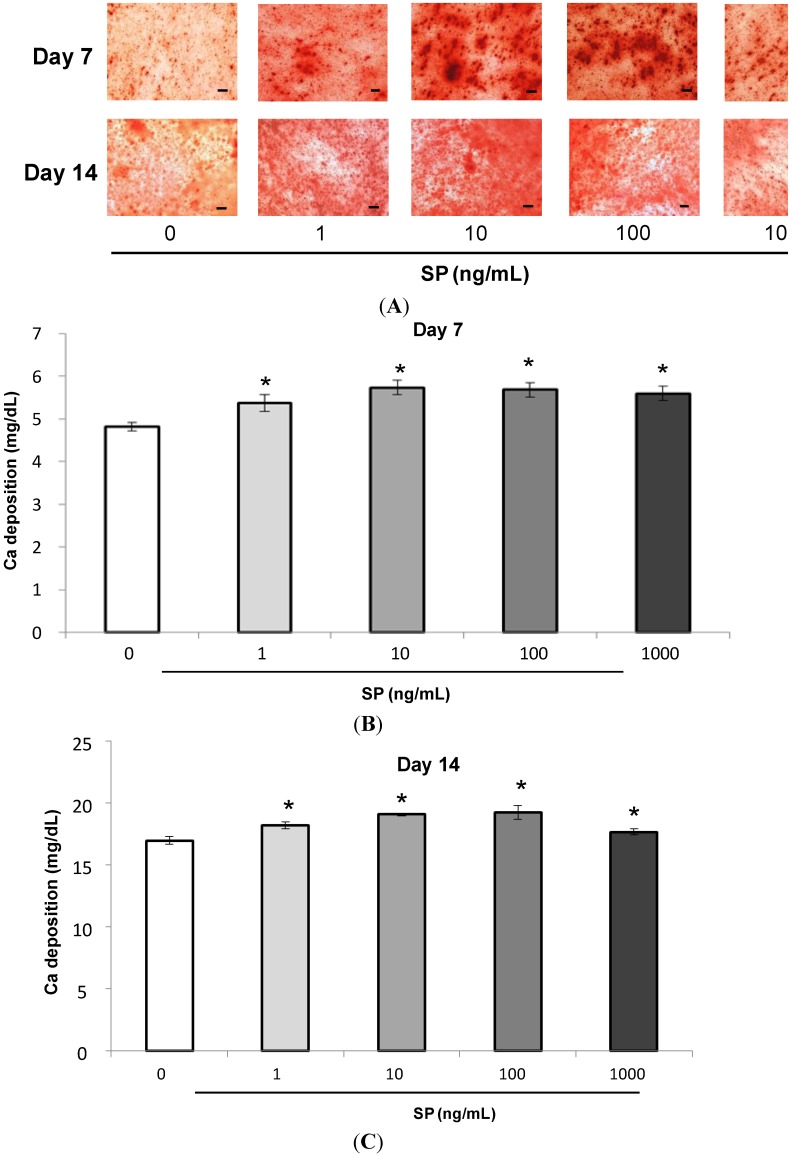
Effect of SP on mineralization. (**A**) Confluent MSCs were stained with Alizarin Red after 7 or 14 days of cultivation in osteogenic medium containing SP (0, 1, 10, 100 or 1000 ng/mL). Scale bar = 100 μm; (**B**,**C**) The extracellular calcium deposition was measured at 7 or 14 days. (**B**) day 7, * *p* < 0.05, *vs.* SP (0 ng/mL); (**C**) day 14, * *p* < 0.05, *vs.* SP (0 ng/mL).

The intensity of Alizarin staining became stronger after addition of SP (Figure 4A, days 7 and 14). The number of calcified nodules stained with Alizarin Red also increased. SP also significantly promoted extracellular calcium deposition at 7 and 14 days (Figure 4B day 7, and Figure 4C day 14, * *p* < 0.05). Additionally, SP promoted the highest intensity of Alizarin Red staining and the most extracellular calcium deposition at a concentration of 10 ng/mL.

#### 2.1.5. Effects of an Extracellular Signal-Related Kinases (ERK) 1/2 Inhibitor on SP-Induced Cell Proliferation and Osteoblastic Differentiation

**Figure 5:**
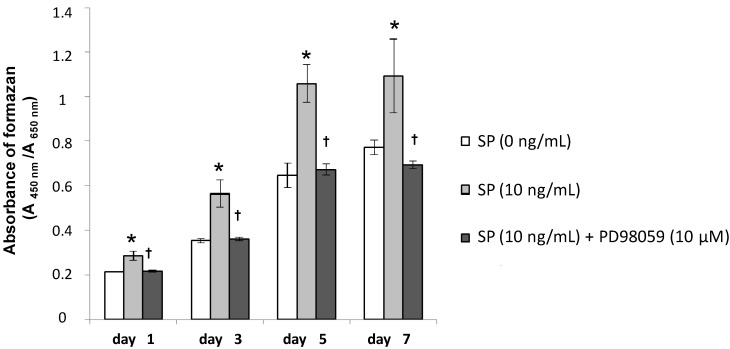
Effect of PD98059 on SP-induced cell proliferation. MSCs were seeded in 96-well plates at 2 × 10^3^ cells/well in normal culture medium. After a 24 h culture for cell adherence, MSCs were cultured in normal culture medium containing SP (0 or 10 ng/mL), or SP (10 ng/mL) with the ERK 1/2 inhibitor PD98059 (10 µM). Cell proliferation was measured on days 1, 3, 5 and 7. * *p* < 0.05, SP (0 ng/mL) *vs.* SP (10 ng/mL), ^†^ *p* < 0.05, SP (10 ng/mL) *vs.* SP (10 ng/mL) with PD98059 (10 µM).

**Figure 6:**
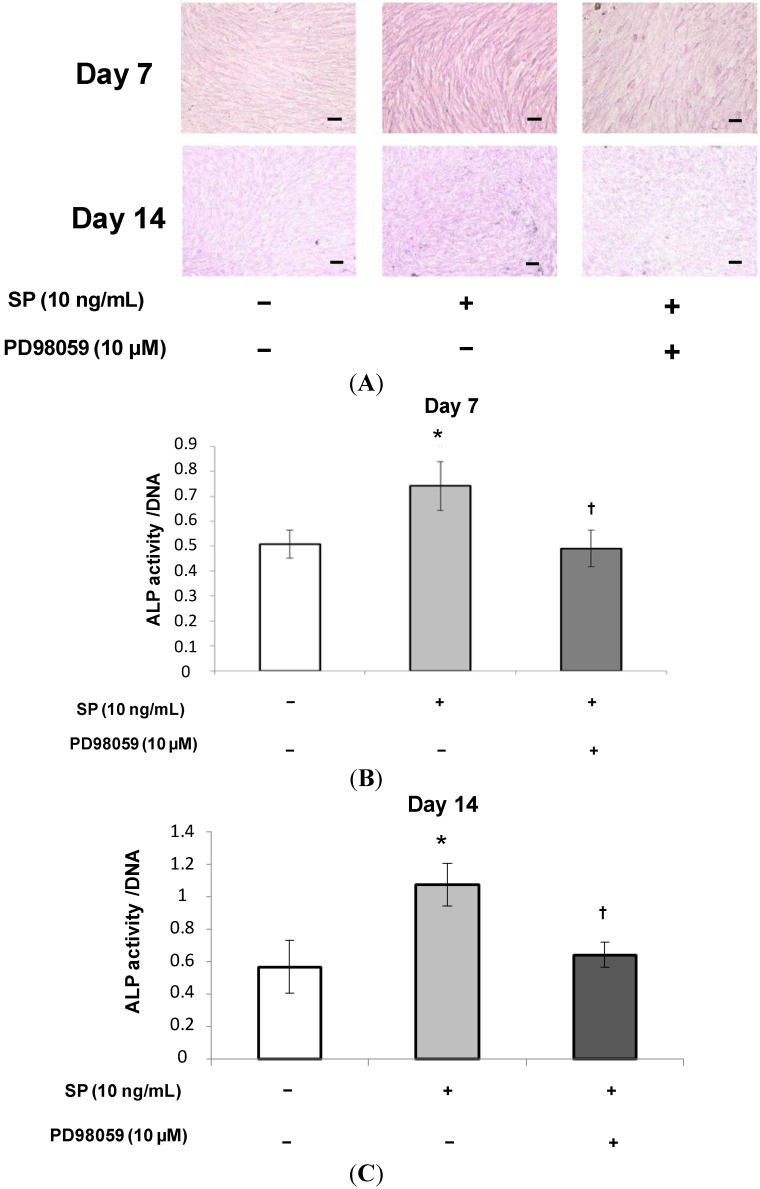
Effect of PD98059 on ALP staining and ALP activity induced by SP. (**A**) Confluent MSCs were stained using an ALP staining kit after 7 or 14 days of cultivation in osteogenic medium containing SP (0 or 10 ng/mL), or SP (10 ng/mL) with the ERK 1/2 inhibitor, PD98059 (10 µM). Scale bar = 100 µm; (**B**,**C**) ALP activity was measured at 7 or 14 days. To normalize ALP activity, the amount of ALP was normalized to the amount of DNA. * *p* < 0.05, SP (0 ng/mL) *vs.* SP (10 ng/mL), ^†^ *p* < 0.05, SP (10 ng/mL) *vs.* SP (10 ng/mL) with PD98059 (10 µM).

**Figure 7:**
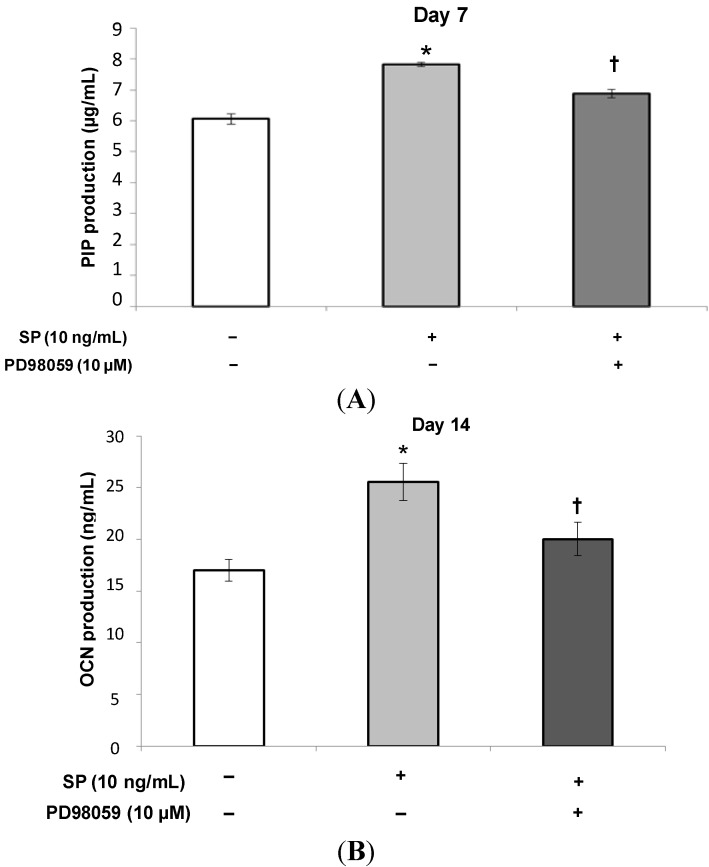
Effect of PD98059 on the production of PIP and OCN induced by SP. After the MSCs reached confluence, the culture medium was replaced with osteogenic medium containing SP (0 or 10 ng/mL), or SP (10 ng/mL) with the ERK 1/2 inhibitor, PD98059 (10 µM) and the cells were cultured for 7 (**A**) or 14 (**B**) days. * *p* < 0.05, SP (0 ng/mL) *vs.* SP (10 ng/mL), ^†^ *p* < 0.05, SP (10 ng/mL) *vs.* SP (10 ng/mL) with PD98059 (10 µM).

**Figure 8:**
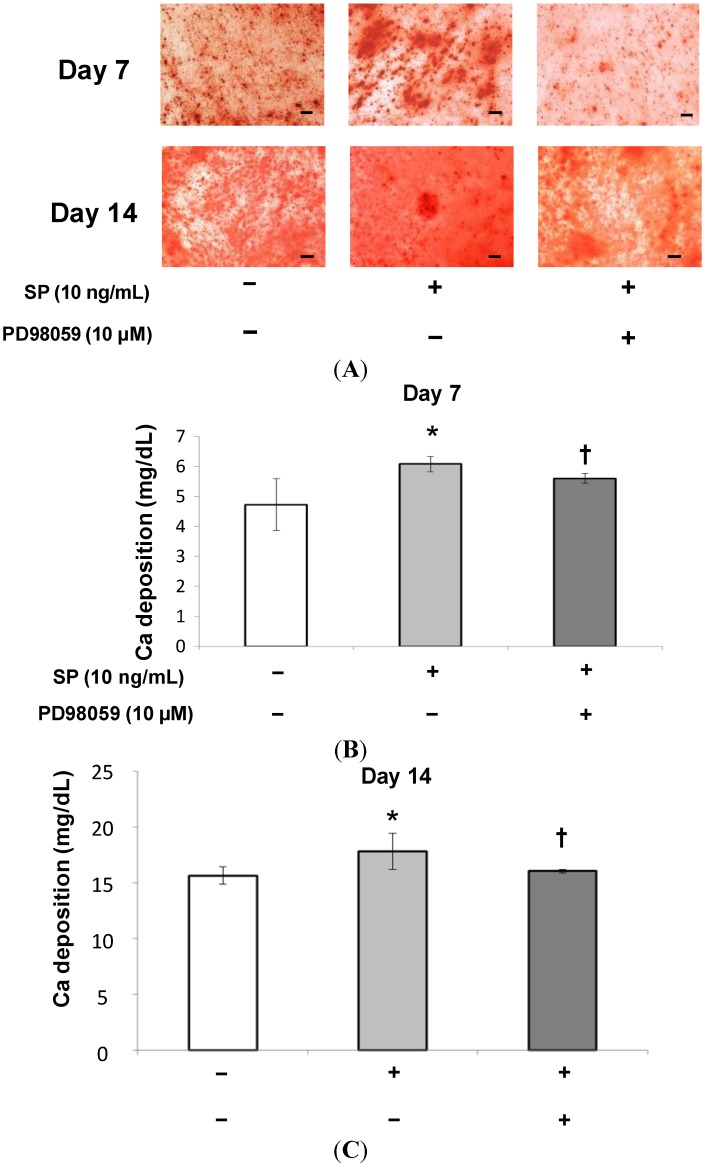
Effect of SP on mineralization. (**A**) Confluent MSCs were stained with Alizarin Red after 7 or 14 days of cultivation in osteogenic medium containing SP (0 or 10 ng/mL), or SP (10 ng/mL) with the ERK 1/2 inhibitor, PD98059 (10 µM). Scale bar = 100 µm; (**B**,**C**) The extracellular calcium deposition was measured at 7 or 14 days. * *p* < 0.05, SP (0 ng/mL) *vs.* SP (10 ng/mL), ^†^ *p* < 0.05, SP (10 ng/mL) *vs.* SP (10 ng/mL) with PD98059 (10 µM).

At 10 ng/mL SP had the strongest effect on cell proliferation and osteoblastic differentiation. Therefore, we added the ERK 1/2 inhibitor, PD98059 (10 μM), to the culture medium containing 10 ng/mL SP to investigate the role of ERK 1/2 in the effects of SP. As shown in Figure 5, cell proliferation significantly decreased in cultures treated with both the inhibitor and SP. Additionally, as shown in Figure 6, Figure 7 and Figure 8, the intensity of ALP staining, ALP activity, PIP production, OCN production, and mineralization (the intensity of Alizarin Red staining and extracellular calcium deposition) significantly decreased in cultures treated with the inhibitor and SP.

### 2.2. Discussion

The results of this study indicate that SP promotes cell proliferation, osteoblastic differentiation, and mineralization of MSCs that contribute to periodontal tissue regeneration. Moreover, an ERK 1/2 inhibitor suppressed these SP-induced effects.

EMD promotes cell proliferation of C2C12 cells [29], a typical pluripotent mesenchymal cell line. EMD also promotes cell proliferation of bone marrow stromal cells that contribute to the regeneration of various tissues [30]. In our previous studies, SP promoted cell proliferation of rat bone marrow stromal cells [18] and PDL fibroblasts [17,19]. SP also promoted cell proliferation of PDL stem cells [20]. In the current study, SP promoted cell proliferation of MSCs. Specifically, SP at a concentration of 10 ng/mL had the strongest stimulatory effect. In the process of periodontal tissue regeneration, the promotion of MSC cell proliferation is essential. Therefore, SP, which promotes cell proliferation of MSCs, contributes to periodontal tissue regeneration.

ALP is a marker of the osteogenic phenotype and indicates the activity of mineralization and hard tissue formation processes [31,32]. EMD promotes ALP activity in osteoblasts [33] and C2C12 cells [29]. However, Guida *et al.* have suggested that EMD inhibits ALP activity in human bone marrow stromal cells [30]. Conversely, SP promotes ALP activity in PDL fibroblasts [19] and rat bone marrow stromal cells [18]. In the current study, SP increased the intensity of ALP staining and ALP activity in MSCs. Therefore, SP stimulates ALP activity and promotes osteoblastic differentiation of human MSCs.

The formation of new bone requires the synthesis of type I collagen [34]. PIP is a precursor of type I collagen and plays an important role in early bone formation. EMD promotes the production and mRNA expression of *type I collagen* [35,36]. SP also promotes the immunohistological expression of type I collagen after injection into tissues with artificial periodontal defects [16]. As shown in Figure 3A, SP promoted the production of PIP in human MSCs. Therefore, it is likely that SP promotes the production of PIP and induces an early stage of osteoblastic differentiation.

OCN is a non-collagenous protein component of bone matrix and a late marker of osteoblastic differentiation [37]. EMD promotes *OCN* mRNA expression in C2C12 cells [29], and enamel matrix proteins (EMPs) extracted from swine promote *OCN* mRNA expression in mouse bone marrow stromal cells [38]. SP also promotes the production of OCN in PDL fibroblasts [19] and PDL stem cells [20]. In our study, SP promoted OCN production in MSCs. Subsequently, SP seemed to induce the differentiation of MSCs into mature osteoblasts, because OCN was secreted during the late stage of bone formation.

We quantified the calcium deposition in cultured MSCs using a biochemical mineralization assay based on Alizarin Red staining and extracellular calcium deposition. Alizarin Red staining is used to ascertain the presence of mineralized nodules formed by cells of osteogenic lineages; these nodules are indicative of bone matrix calcification.

Alizarin Red staining has also been employed to detect the mineralization of MSCs. EMD promotes the mineralization of rat bone marrow stromal cells [39,40]. The results of the current study suggest that SP promotes both the number of mineralized nodules stained with Alizarin Red, as well as the extracellular calcium deposition in MSCs. Mineralization is essential for regenerating periodontal tissue lost by periodontal disease. These findings suggest that SP promotes the mineralization of the extracellular matrix in MSCs and plays a role in the regeneration of periodontal tissue.

ERK 1/2 is one of the MAPK family members and regulates both cell proliferation and osteoblast differentiation. It has been suggested that EMD and the other amelogenin peptides can regulate cellular functions through the ERK 1/2 signaling pathway [41,42]. We speculated that cell proliferation and osteoblastic differentiation of human MSCs are regulated by the ERK 1/2 signaling pathway as well as EMD. Therefore, using the ERK 1/2 inhibitor, PD98059, we examined the molecular mechanism by which SP affects cell proliferation and osteoblastic differentiation in human MSCs. The concentration of the inhibitor (10 µM) was set according to a previous report [41]. As shown in Figure 5, Figure 6, Figure 7 and Figure 8, the inhibitor significantly suppressed cell proliferation and osteoblastic differentiation induced by SP. Therefore, we propose the hypothesis that SP promotes cell proliferation and osteoblastic differentiation thorough the ERK 1/2 signaling pathway. However, the detailed mechanism was not completely revealed. We think it is necessary to examine the mechanism by which SP induces cell proliferation and osteoblastic differentiation using other inhibitors of MAPKs involved in cell proliferation and differentiation, such as p38 and JNK. Thus, in future studies we will investigate more signaling pathways.

In our previous study, SP had the strongest effect on cell proliferation and osteoblastic differentiation of PDL fibroblasts and PDL stem cells at a concentration of 100 ng/mL. In the current study, 10 ng/mL SP had the strongest effect on cell proliferation and osteoblastic differentiation of human MSCs. We suggest that at a high concentration of SP (100 ng/mL) cell proliferation and osteoblastic differentiation are induced in PDL fibroblasts and PDL stem cells, which directly construct periodontal tissues. However, a lower SP concentration (10 ng/mL) is the optimum for MSCs to induce indirect participation in periodontal tissue regeneration. Thus it appears that different cells have different optimal concentrations. In the present study, we found for the first time that the optimal concentration of SP is different depending on the type of cell. EMD has various effects on cells at various stages of cell proliferation and cell differentiation [43,44]. Thus, we speculate that SP also has various effects depending on cell type and cell maturation stage. However, the reasons behind the different optimal concentrations are not clear, yet. Hence, we intend to examine this question in future research.

In the study of periodontal tissue regeneration, EMD and recombinant amelogenin peptides are used *in vitro* and *in vivo*. EMD is derived from animal tissue. Moreover, EMD has been reported to be antigenic and to induce the production of anti-EMD antibodies. In contrast, SP can be artificially synthesized. Because its molecular mass is lower than that of EMD and other recombinant amelogenin peptides, it is less likely to elicit an immunological response. Therefore, we suggest that SP is advantageous because it can be artificially synthesized and is not likely to induce antibodies in the host. These benefits are very useful for clinical treatments.

Miron *et al.* have suggested that EMD promotes cell clustering via connexin 43 molecules and that this cell clustering involves osteoblastic differentiation and mineralization of human osteoblasts [45]. In Figure 4A, SP appeared to cause cell clustering and calcified nodules seemed to form along with the cell clustering. Thus, we suggest that similarly to EMD, SP forms cell clustering and may involve mineralization.

## 3. Experimental Section

### 3.1. Preparation of Synthetic Oligopeptide and Cell Culture

We synthesized SP based on the sequence, WYQNMIR, by traditional solid-phase peptide synthesis in conjunction with the “tea-bag” methodology using Boc/benzyl-based chemistry, as previously described [11]. SP was prepared at the concentrations of 0, 1, 10, 100 and 1000 ng/mL in culture medium. Human MSCs (Yub625) were purchased from the Riken Bioresource Center (Kobe, Japan). The origin of human primary MSCs was human cartilage. MSCs have high proliferative capacity and the potential of osteoblast differentiation. MSCs were cultured in normal culture medium in the presence of 10% fetal bovine serum (Gibco BRL, Life Technology, Grand Island, NY, USA), 500 U/mL penicillin and 500 µg/mL streptomycin (Nacalai Tesque, Kyoto, Japan). MSCs were seeded onto T75 culture dishes (Falcon BD, Franklin Lakes, NJ, USA) and incubated at 37 °C in 5% CO_2_. MSCs at passage 5 (P5) were used for further experiments. In the osteoblastic differentiation assay, we used osteogenic medium containing 50 µM l-ascorbic acid 2-phosphate (Nacalai Tesque, Kyoto, Japan), 10 mM β-glycerophosphate (Wako Pure Chemical Industries Ltd., Tokyo, Japan), and 10 nM dexamethasone (Wako Pure Chemical Industries Ltd., Osaka, Japan).

### 3.2. Cell Proliferation Assay

MSCs were seeded in 96-well plates at 2 × 10^3^ cells/well in normal culture medium. After a 24 h culture for cell adherence, the medium was replaced with normal culture medium containing SP (0, 1, 10, 100 or 1000 ng/mL) and MSCs were incubated for 1, 3, 5 and 7 days. The number of viable cells at each time point was determined by measuring the amount of formazan generated in six wells per group using the Cell Count Reagent SF (Nacalai Tesque, Kyoto, Japan). The absorbance of formazan was measured at 450 nm, and the data were analyzed with the SoftMax^®^ Pro Microsuede Data Acquisition and Analysis software (Molecular Devices, Sunnyvale, CA, USA).

### 3.3. Alkaline Phosphatase (ALP) Staining and Measurement of ALP Activity

MSCs were seeded at 4 × 10^4^ cells/well in 24-well plates and were cultured to confluence in normal culture medium. The medium was then replaced with osteogenic medium containing SP (0, 1, 10, 100 or 1000 ng/mL). After 7 or 14 days, MSCs were washed with PBS and fixed. ALP staining was performed using an Alkaline Phosphatase kit (Sigma-Aldrich, St. Louis, MO, USA) according to the manufacturer’s protocol.

MSCs were cultured for 7 or 14 days, and then washed with PBS and lysed with 300 µL of 0.2% Triton X-100 (Sigma-Aldrich). ALP activity was measured by a 1-step pNPP substrate (Pierce Biotechnology, Inc., Rockford, IL, USA). The DNA content was measured using the PicoGreen dsDNA Assay kit (Invitrogen, Paisley, UK). To normalize ALP activity, the amount of ALP was normalized to the amount of DNA in the cell lysate. The data were analyzed with the SoftMax^®^ Pro software.

### 3.4. Measurement of Procollagen Type 1 C-Peptide (PIP) and Osteocalcin (OCN) Production

MSCs were seeded at 4 × 10^4^ cells/well in 24-well plates and were cultured to confluence in normal medium. The medium was replaced with osteogenic medium containing SP (0, 1, 10, 100 or 1000 ng/mL) and MSCs were cultured for 7 or 14 days. The culture supernatant was collected, and then PIP and OCN levels were measured with a PIP EIA kit (Takara Bio Inc., Otsu, Japan) and Gla type Osteocalcin EIA kit (Takara Bio Inc., Otsu, Japan).

### 3.5. Mineralization Assay (Alizarin Red Staining and Measurement of Extracellular Calcium Deposition)

MSCs were seeded at 4 × 10^4^ cells/well in 24-well plates and were cultured to confluence in normal medium. The medium was then replaced with osteogenic medium containing SP (0, 1, 10, 100 or 1000 ng/mL) and MSCs were cultured for 7 or 14 days. The medium was removed, the cells were washed with PBS, and the MSCs were fixed in 70% ethanol for 10 min at −20 °C. MSCs were stained with a solution of 1% Alizarin Red S (Wako Pure Chemical Industries Ltd., Osaka, Japan) for 3 min at room temperature and washed three times with distilled water.

Extracellular calcium deposition was measured after the calcium was dissolved in 10% formic acid (Nacalai Tesque, Kyoto, Japan). The calcium was quantified using a Calcium E-test kit (Wako Pure Chemical Industries Ltd., Osaka, Japan) according to the manufacturer’s protocol. The reaction product absorbance was measured at 610 nm and analyzed with the SoftMax^®^ Pro software.

### 3.6. Effect of Extracellular Signal-Related Kinases (ERK) 1/2 Inhibition on Cell Proliferation and Osteoblast Differentiation

In this study, we treated MSCs with SP (0 to 1000 ng/mL) to investigate the optimal concentration. We determined the optimal concentrations at which SP had the strongest effect on cell proliferation and osteoblast differentiation. Using PD98059, an ERK 1/2 inhibitor, we investigated the role of ERK 1/2 in SP-induced (optimal concentration) effects on MSCs (Sigma-Aldrich, St. Louis, MO, USA). MSCs were treated with culture medium containing 10 µM PD98059 in the cell proliferation and osteoblast differentiation assays.

### 3.7. Statistical Analysis

In the present study, six wells were prepared for the cell proliferation assay, and three wells were prepared for the remaining experiments; each experiment was repeated three times. The statistical analysis was performed using IBM SPSS Statistics Ver. 17 (IBM, Chicago, IL, USA). One-way analysis of variance (ANOVA) followed by Tukey’s *post hoc* test was used to determine significance. *p* values < 0.05 were considered significant.

## 4. Conclusions

In conclusion, the present study suggests that SP promotes cell proliferation, osteoblast differentiation, and mineralization in human MSCs. These results indicate that SP would be useful for periodontal tissue regeneration, because the promotion of cell proliferation and differentiation in MSCs is essential for this process. Moreover, our results suggest that SP promotes cell proliferation and osteoblast differentiation through the ERK 1/2 pathway. These results show for the first time the effects of SP on human MSCs. However, the molecular mechanisms of SP are not clear, yet, and it is necessary to investigate the detailed molecular mechanisms of SP in future studies.
